# Evaluation of Cardiac Function in Patients with Thalassemia Intermedia

**Published:** 2013-01-22

**Authors:** NM Nouri, M Naderi, S Rajaie, A Dorgalaleh, Sh Tabibian

**Affiliations:** 1 Children and Adolescent Hygiene Research Center (CAHRC) & Clinical Research Development Center (CRDC), Ali ebn-e Abitaleb (AS) teaching hospital,Zahedan University of Medical Sciences, Zahedan, Iran.; 2 Children Clinical Research Development Center (CCRDC), Hormozgan University of Medical Sciences, Bandar Abbas, Iran.; 3 Allied Medicl School, Departman of Hematology, Tehran University of Medical Science, Tehran, Iran.

**Keywords:** Heart failure, Anemia, beta-Thalassemia

## Abstract

**Background:**

Thalassemia intermedia is a variety of beta thalassemia which shows clinical symptoms somewhere between asymptomatic carriers and thalassemia major. Cardiac dysfunctions due to chronic anemia and hemosiderosis are the major causes of death in these patients. The purpose of this study is to evaluate cardiac function in these patients by echocardiography.

**Materials and Methods:**

This case-control study was conducted on 22 thalassemic patients (mean: 16.5±5.8 years) and 66 healthy individuals (mean:16.07± 2.9years) as a control group from January 2007 to July 2008. There was no sign of cardiac involvement by physical examination, chest x-ray and ECG in patients. Echocardiographic parameters were measured in groups, and finally data was analyzed by SPSS software.

**Results:**

The mean of left ventricular myocardial performance index (MPI) (P-value=0.0001) and left ventricular mass index (LVMI) (P-value=0.0001) have statistically significant difference. Mean of interventricular septal dimension in diastole (IVSD), left ventricular posterior wall thickness in diastole (LVPWD), interventricular septal dimension in systole (IVSS) and left ventricular posterior wall dimension in systole (LVPWS) were also statistically significant with a P-value of, 0.002, 0.001, 0.01, 0.003, respectively. Aortic Pre-ejection period/Ejection time (PEP/ET) (P-value=0.009), ejection fraction (EF) (P-value=0.019), fractional shortening (SF) (P-value=0.041), left ventricular isovolumetric contraction time (ICT) (P-value=0.0001) and left ventricular isovolumetric relaxation time (IRT) (P-value=0.0001) were statistically significant. Mean of right ventricular MPI (P-value=0.0001) and IRT (P-value=0.0001) were also significantly different between two groups. Others echocardiographic parameters were not statistically significant (P-value>0.05).

**Conclusion:**

Heart failures are earlier affected thalassemia intermedia patients compared with control group.

## Introduction

Thalassemia is one of the most common hematologic disorders in human beings. This disease is prevalent in Mediterranean region, India, Burma, north of China, up to regions in Pacific Islands. Thalassemia is of two major types namely Alpha and Beta in which the alpha and beta globin chains are involved. 

The main feature of this anemia syndrome concerns the deficiency or the lack of one or more globin chains (1, 2). Beta-thalassemia intermedia is a kind of beta thalassemia which shows clinical symptoms somewhere between asymptomatic carriers and thalassemia major. By definition, the patients who spontaneously maintain hemoglobin at or above 7g/dl, even at the price of bone marrow hyperplasia, characterized as thalassemia intermedia (2, 3).

The main reasons of early death in such patients concern the cardiac complications such as systolic and diastolic disorders caused by hemosiderosis (4, 5). The studies conducted so far indicated that the end-systolic and end-diastolic dimension index and also the whole heart index and systolic dimension of thalassemia intermedia patients have a significant increase compared with control group. It happens while the patients did not receive blood transfusion and did not show any clinical symptoms of cardiac disorder (6). 

Two factors have a major role in the heart failure in thalassemia major patients, the increase in cardiac output and vascular complications (7).

In the absence of regular treatment of thalassemia intermedia patients the diastolic performance of the left ventricle is maintained stable, while the pressure of pulmonary arteries continues to rise which is the main cause of death in these patients (8). It seems that iron plays a significant role in pathophysiology of heart failure of thalassemia major, but in thalassemia intermedia it assumes that cardiac output is the major cause of heart failure (9). Since no study has been reported about cardiac failure of such patients in Iran, and the studies in other parts of the world are not plenty either, the present paper investigated the heart performance of these patients by means of echocardiography.

## Materials and Methods

This case-control study conducted on 22 patients with thalassemia intermedia in the range of 8to25 years old that underwent treatment in pediatric ward of Ali Ebn-e Abitaleb (AS) hospital of Zahedan city, from January 2007 until July 2008. The patients were initially interviewed, clinically examined, and then underwent chest radiography and electrocardiography. 

Those participants who did not have any obvious cardiac disorder and patients who suffered from high blood pressure, metabolic, endocrine or kidney disorders or used to take heart drugs, or Hb<8g/dl were excluded from the study.

Sixty-six healthy children with the same age and gender that referred to our hospital for routine checkup were selected as control group B. Written consent was obtained from each participant and the study was approved by the medical ethics committee of Zahedan University of Medical Science. Both groups underwent echocardiography test by 2D, M-mode, and Doppler methods and parameters such as Aorta/Left atrium diameter (AO/LA), Shortening fraction (SF), Ejection fraction (EF), Left ventricular end-systolic dimension (LVESD), Left ventricular end-diastolic dimension (LVEDD), Left ventricular posterior wall dimension in systole (LVPWS), Interventricular septal dimension in systole (IVSS), Left ventricular posterior wall dimension in diastole (LVPWD), Interventricular septal dimension in diastole (IVSD), Pre-ejection period/Ejection time (PEP/ET), Ejection time (ET), Pre-ejection period (PEP), Left ventricular mass index (LVMI), Myocardial performance index (MPI), E/A velocity ratio (E/A), Peak A velocity (Peak A), Peak E velocity (Peak E), Deceleration time (DT), Deceleration slope (DS), Acceleration time (AT), Isovolumic relaxation time (IRT), and Isovolumic contraction time (ICT) were measured in both groups.

**Statistical analysis**

Data analysis was performed by SPSS software (version 18), χ^2^ (Chi-square) test and also Fisher Exact test were used as needed. Moreover, for comparison between two groups, we used t-test after determining the normality of data distribution by nonparametric Kolmogorov-Smirnov test. Pearson correlation coefficients also were used to determine the correlation between quantitative variables. A P-value of P-value<0.05 were considered as statistically significant.

## Results

The mean of the age in the group A was 16.5±5.8, while in the group B it was 16.07± 2.9 (P-value=0.6). Comparison of age and gender revealed no significantly difference between two groups of case and control (P-value>0.05).

The average of hemoglobin and hematocrit in group A and B were 7.5 g/dl (24%) and 13 g/dl (42%), respectively. Table I represents the echocardiographic parameters of left ventricle in patient and control groups. The mean of the myocardial performance index (MPI) of the right and left ventricles was statistically significant between group A and B (P-value=0.0001). The mean of left ventricular myocardial index (LVMI) was also statistically significant between these two groups of case and control (P-value=0.0001). The mean of the aorta pre-ejection period (PEP) and ejection time (ET) in the group A (P-value=0.19) and group B (P-value=0.034), respectively didn’t show any statistically difference, while the difference between mean of aorta PET/ET was statistically significant (P-value=0.009). 

The mean of interventricular septal dimension in diastole (IVSD) of the group A and group B (P-value=0.002), the mean of left ventricular posterior wall dimension in diastole (LVPWD) (P-value=0.001), the mean of interventricular septal dimension in systole (IVSS) (P-value=0.01), and the mean of left ventricular posterior wall dimension in systole (LVPWD) (P-value=0.003), all indicated significant difference. The mean of left ventricular end-diastolic dimension, left ventricular end-systolic dimension in group A (P-value= 0.78) and group B (P-value=0.49), did not show any significant difference. 

The mean of ejection fraction (P-value=0.019) and the mean of fraction shortening (P-value=0.041) in both groups was statistically significant. The mean of aorta (AO) diameter and left atrium (LA) diameter in diastole (P-value=0.43) and (P-value=0.09), respectively, and the LA/AO (P-value=0.32) in the group A and group B did not show statistically significant difference. The mean of ICT and IRT of the left ventricle in the group A and group B showed no statistically significant difference (P-value=0.0001). 

The mean of acceleration time (AT) of mitral valve (P-value=0.43), deceleration slope (DS) (P-value= 0.54), deceleration time (DT) (P-value=0.88), peak E velocity (P-value=0.74), peak A velocity (P-value= 0.34) and E/A velocity (P-value=0.34) in both groups did not show statistically significant difference. Table II displays the right ventricle echocardiography parameters of the patient and control groups. The mean of right ventricle IRT in the group A and group B was statistically significant (P-value=0.0001), while the mean of right ventricle ICT between these two groups did not show any statistically significant difference (P-value= 0.59). 

The mean of pulmonary ET, PEP, and PEP/ET in the group A and group B was not statistically significant. The mean of AT, DS, DT, peak E velocity and E/A of tricuspid valve in both groups was not statistically significant, while the mean of peak A velocity in group A and B was statistically different (P-value=0.04). 

**Table I:** Comparsion between two groups of case and control at the beginning of study

	group A	group B	P-value
Age (mean)	16.5±5.8 years	16.07± 2.9 years	P>0.05
Male	54%	52%	P>0.05
Female	46%	48%	P>0.05
Weight (mean)	35.65 ± 11.36 kg	54.01± 5.84 kg	P=0.01
Height (mean)	137.8 ± 17.86 cm	152.± 13.52 cm	P= 0.0001

**Table II:** Echocardiographic parameters of left ventricle of the patient and control groups

Parameter*	Thalassemia Intermedia group (n=22)	Control group (n=66)	P-value
MPI	0.14±0.61	0.06±0.4	**0.0001**
LVMI	40.86±.110.5	32.60±76.53	**0.0001**
PEP	9.90±93.31	9.46±90.85	**0.19**
ET	25.45±250.8	20.76±07/262	**0.034**
PEP/ET	0.03±0.36	0.04±0.34	**0.009**
IVSD (mm)	1.76±7.29	1.02±6.44	**0.002**
LVPWD (mm)	1.4±4.61	0.49±3.99	**0.001**
LVPWS (mm)	1.50±4.72	0.48±4.13	**0.003**
LVEDD (mm)	6.93±48.72	5.98±48.31	**0.78**
LVESD (mm)	5.36±31.78	7.6±32.93	**0.49**
EF (%)	7.12±61.56	6.63±65.46	**0.019**
SF (%)	5.26±33.68	4.94±36.17	**0.041**
AO(mm)	4.08±24.26	2.95±23.63	**0.42**
LA (mm)	3.81±29.45	3.81±27.89	**0.09**
AO/LA (mm)	0.17±1.22	0.17±1.18	**0.32**
ICT (msec)	18.78±43.68	11.13±28.10	**0.0001**
IRT (msec)	16.64±110.09	15.34±96.25	**0.0001**
AT (msec)	16.33±61.86	9.67±78.10	**0.43**
DS (msec)	31.79±122.31	35/23±118.56	**0.54**
DT (msec)	18.78±126.09	18.69±125.43	**0.88**
Peak E (m/sec)	16.76±102.04	24.61±103.85	**0.44**
Peak A (m/sec)	12.70±46.65	15.19±61.31	**0.34**
E/A (m/sec)	**0.23** **±** **1.59**	**0.73** **±** **1.94**	**0.34**

MPI: Myocardial performance index; LVMI: Left ventricular mass index; PEP: Pre-ejection period; ET: Ejection time; PEP/ET: Pre-ejection period/Ejection time; IVSD: Interventricular septal dimension in diastole; LVPWD: Left ventricular posterior wall dimension in diastole; IVSS: Interventricular septal dimension in systole; LVPWS: Left ventricular posterior wall dimension in systole; LVEDD: Left ventricular end-diastolic dimension; LVESD: Left ventricular end-systolic dimension; EF: Ejection fraction; SF: Shortening fraction; AO: Aorta diameter;LA: Left atrium diameter; AO/LA: Aorta/Left atrium diameter; ICT: Isovolumic contraction time; IRT: Isovolumic relaxation time; AT: Acceleration time; DS: Deceleration slope; DT: Deceleration time; Peak E: Peak E velocity; Peak A: Peak A velocity; E/A: E/A velocity ratio

**Table III:** Echocardiographic parameters of right ventricle of the patient and control groups

Parameter*	Thalassemia Intermedia group (n=22)	Control group (n=66)	P-value
MPI	0.13±0.63	0.52±0.08	**0.0001**
IRT (msec)	20.92±131.13	12.97±107.02	**0.0001**
ICT (msec)	20.98±38.44	15.12±36.33	**0.59**
PEP(msec)	11.62±92.1	8.24±91.87	**0.88**
ET (msec)	29.81±251.9	23.17±260.67	**0.145**
PEP/ET	0.06±0.36	0.03±0.34	**0.086**
AT (msec)	23.04±67.68	13.84±67.63	**0.95**
DS (msec)	27.90±121.95	36.86±120.36	**0.85**
DT (msec)	26.04±125.86	22.19±134.55	**0.12**
Peak E (m/sec)	15.90±63.44	15.29±61.27	**0.56**
Peak A (m/sec)	16.36±50.51	13.1±43.57	**0.04**
E/A (m/sec)	**0.37** **±** **1.33**	**0.29** **±** **1.44**	**0.12**

MPI: Myocardial performance index; IRT: Isovolumic relaxation time; ICT: Isovolumic contraction time; PEP: Pre-ejection period; ET: Ejection time; PEP/ET: Pre-ejection period/Ejection time; AT: Acceleration time; DS: Deceleration slope; DT: Deceleration time; Peak E: Peak E velocity; Peak A: Peak A velocity; E/A: E/A velocity ratio.

## Discussion

Beta-thalassemia intermedia is a subcategory of beta thalassemia that its clinical manifestations vary from thalassemia major to asymptomatic carriers. The patients with hemoglobin over 10gr/dl require no blood transfusion, and since they show no clinical traits, they may grow up to adulthood. Another group of patients have hemoglobin level of 6gr/dl and therefore, require blood transfusion, and they may display clinical feature such as bone deformity, arthritis, bone pain, progressive splenomegaly, growth failure, ankle ulcers and cardiovascular disorders. Cardiomyopathy is regarded as the main cause of mortality among these patients (2, 7). Thus, this study aimed to ascertain heart performance of these patients by means of echocardiography.

Our study revealed that, MPI of the right and left ventricles of patients with thalassemia intermedia was higher than healthy individuals similar to the findings of Bosie et al (10).

The increase of LVMI in the patients of our study was similar to the patients of Vaccari which is regarded as the main factor in the cardiac compliance of these patients that could be a reason for the high frequency of occurrence of diastolic dysfunction in such patients (6). The increase of LVMI was also observed by Bosie and Ocal (10, 11)

We also found that PEP in the left side shows a significant increase. The increase of PEP/ET indicates early changes of diastole performance in ventricles. The mean of IVSD of patients group was higher than control group which is in agreement with the findings of Bosie et al (11). 

The left ventricular end diastolic dimension in our study was similar to Aessopos and Bosie studies but the left ventricular end systolic dimension did not show this increase, which again agrees with Aessopos study (8). The mean of left ventricular posterior wall dimensions in the patients increased which matches with Aessopos study (4). Our results showed statistically significant difference between mean of EF in the patient group compared with the control group that was similar to Aessopos study (8). Similar to the studies of Bosie and Vaccari, the FS of the patients group was decreased which itself is a sign of left ventricle dysfunction (6, 8, 10). 

IRT increase of the right ventricle is due to the disorder in the filling of right ventricle in thalassemia patients. IRT increase of the left ventricle which is a diastolic performance index has been reported in various studies on thalassemia patients that are due to disorder during rest time of the ventricle caused by iron deposits (12, 14). It is considered as the gradual cause for restrictive cardiomyopathy which is the earliest symptom of diastolic performance disorder of left ventricle (12, 13). Low systolic and diastolic blood pressure and high heart rate of asymptomatic patients have also been mentioned in Vaccari and Bosie studies, the cause of low blood pressure in such patients can be attributed to decrease of systemic vascular resistance (6, 8, 10). Increase volume load can also be attributed to Frank Starling mechanism which accelerates heart rate (14). The study of Ismaeel et al showed that the evaluation of the right ventricle performance will be beneficial In order to localize the pathological effects of cardiomyopathy in thalassemia intermedia patients (15).

## Conclusion

It can be concluded that systolic and diastolic performance of thalassemia intermedia patients is affected in comparison with control group due to parameters including MPI, LVMI, IRT, EF, FS, IVSD and LVPWD. For more evaluation, more quantitative and comprehensive research suggested by evaluation of specific effective factors to psycho-social health of these patients.
